# *“Feel the need to prepare for Armageddon even though I do not believe it will happen”*: Women Veterans’ Firearm Beliefs and Behaviors during the COVID-19 Pandemic, Associations with Military Sexual Assault and Posttraumatic Stress Disorder Symptoms

**DOI:** 10.1371/journal.pone.0280431

**Published:** 2023-02-10

**Authors:** Lindsey L. Monteith, Christin N. Miller, Evan Polzer, Ryan Holliday, Claire A. Hoffmire, Christe’An D. Iglesias, Alexandra L. Schneider, Lisa A. Brenner, Joseph A. Simonetti

**Affiliations:** 1 VA Rocky Mountain Mental Illness Research, Education and Clinical Center for Suicide Prevention, Aurora, Colorado, United States of America; 2 Department of Psychiatry, University of Colorado Anschutz Medical Campus, Aurora, Colorado, United States of America; 3 Department of Physical Medicine and Rehabilitation, University of Colorado Anschutz Medical Campus, Aurora, Colorado, United States of America; 4 Department of Neurology, University of Colorado Anschutz Medical Campus, Aurora, Colorado, United States of America; 5 Division of Hospital Medicine, University of Colorado Anschutz Medical Campus, Aurora, Colorado, United States of America; Uniformed Services University of the Health Sciences, UNITED STATES

## Abstract

**Aims:**

Firearm purchasing increased within the U.S. during the coronavirus disease 2019 pandemic. While rates of firearm ownership and suicide are elevated among women Veterans compared to women non-Veterans, no studies have examined if and how firearm beliefs and behaviors changed among women Veterans during the pandemic. We examined women Veterans’ changes in firearm beliefs and engagement in firearm behaviors during the early pandemic era.

**Method:**

3,000 post-9/11 era women Veterans were invited to participate in a survey. 501 respondents (May-December 2020) comprised the sample for this concurrent nested mixed-method analysis. Thematic analysis and log-binomial regression were used.

**Results:**

13.88% (*n* = 69) of women Veterans in our sample reported changes in their firearm beliefs; 22.15% (*n* = 109) reported engaging in firearm behaviors. The most prevalent reported behaviors were making household firearms more accessible (16.13%) and purchasing ammunition (11.97%). Smaller percentages reported carrying a firearm more frequently (6.71%), loading previously unloaded firearms (5.69%), or purchasing a firearm (4.24%). Thematic analysis suggested firearm behaviors were likely driven by a perceived increased need to protect oneself, family, and property due to: (1) uncertainties brought on by the pandemic; (2) pandemic-related threats necessitating self-defense, preparedness, and self-sufficiency; (3) political, social, and racial unrest and protests. PTSD symptom severity and military sexual assault history were associated with higher prevalence of changes in firearm beliefs and engagement in firearm behaviors during the pandemic.

**Discussion:**

Consideration of women Veterans’ prior experiences and pandemic-related factors may be necessary to contextualize firearm discussions and inform future research. Given associations of military sexual assault and PTSD symptoms with firearm beliefs and behaviors, it may be crucial to ensure that such discussion are trauma-informed.

## Introduction

Within the United States, from 2001 to 2019, the age-adjusted suicide rate for women Veterans increased by 68.7%, from 9.1 to 15.4 per 100,000 [1]. During this time, use of firearms as a method of suicide increased by 12.8% among women Veterans, with 49.8% of those who died by suicide in 2019 using a firearm [2]. Moreover, suicide risk is pronounced among younger Veterans [2], many of whom served in the post-9/11 era. As a result, there have been calls to increase understanding of suicide risk and prevention among women Veterans [3–5], with a focus on firearm-related suicide prevention [6].

Access to firearms, whether personally owned or owned by another household member, is associated with increased risk for suicide [7, 8]. In addition, among those with firearms, specific methods of firearm storage are associated with suicide risk. For example, storing firearms in ways that makes them more readily accessible is more common among individuals who die by suicide [9]. Among military samples, storing loaded firearms at home and carrying a firearm outside of military duty are also associated with increased risk of suicide [10]. For these reasons, understanding factors driving firearm access and unsafe firearm storage (e.g., loaded, unlocked) is critical to Veteran suicide prevention.

Researchers have noted concern regarding increased access to firearms during the coronavirus disease 2019 (COVID-19) pandemic, including how such access could heighten suicide risk [11–14]. Indeed, since the pandemic began, firearm sales in the U.S. have substantially increased [15, 16]. Schleimer and colleagues [17] estimated that 4.3 million more firearm sales than expected occurred during the early months of the pandemic (i.e., March through July 2020). More recently, Miller and colleagues [18] conducted a probability-based online survey and estimated that, from 1/1/2019 until 4/26/2021, 2.9% of U.S. adults became new firearm owners. Notably, females represented half of new firearm owners during that time [18]. In a similar vein, firearm-related background checks increased by 40% in March 2020 –this increase appears to be larger in magnitude than firearm purchases associated with other major national events (e.g., twice as large as firearm-related background checks following 9/11) [19].

These recent changes in firearm access and behaviors are concerning when considering other experiences during the pandemic which could also increase risk for suicide. For instance, during the pandemic, there were increases in anxiety and psychosocial stressors, such as financial worry, occupational instability, school closures, and domestic violence; concurrently, there was decreased access to in-person social support and in-person healthcare appointments, which are important sources of support and coping in the midst of stressful or traumatic experiences [20–23]. Average resilience among U.S. adults decreased during the initial weeks of the COVID-19 pandemic [23].

In spite of this, sparse research has examined whether firearm access has changed among specific populations known to be at elevated risk for suicide, such as women Veterans. Understanding the extent to which women Veterans may have changed their firearm beliefs and engaged in firearm behaviors (e.g., acquired firearms, increased the accessibility of firearms) may facilitate understanding of modifiable suicide risk factors in this population. Further, elucidating specific factors that are associated with changes in firearm beliefs and behaviors could help with understanding how to inform firearm-related suicide prevention efforts in this population in the pandemic’s aftermath.

### Aims

We sought to address these knowledge gaps by examining whether and how women Veterans’ firearm-related beliefs and behaviors have changed since the COVID-19 pandemic began. First, we examined the extent to which women Veterans reported changes in their firearm-related beliefs and engaged in different firearm behaviors. Next, we explored how women Veterans’ firearm beliefs changed during the pandemic and examined women Veterans’ reasons for engaging in firearm behaviors during the pandemic. Finally, we examined factors associated with women Veterans’ changes in firearm-related beliefs and engagement in firearm behaviors, using our qualitative findings to guide the quantitative analyses conducted. We addressed these aims by analyzing survey data, including open-ended free-text responses, that we collected from post-9/11 era women Veterans at the beginning of the COVID-19 pandemic (May through December 2020).

## Method

### Participants and procedures

Data for this manuscript were collected between May through December of 2020 as part of a broader study aiming to understand factors associated with firearm access among post-9/11 women Veterans [24]. Inclusion criteria consisted of being a woman Veteran, 18 to 89 years of age, and having at least one post-9/11 deployment. Veteran status was defined per the *Title 38* definition captured in the VA/DoD Identity Repository (VADIR) [25]. Eligible women had separated from military service, were enrolled in Veterans Health Administration (VHA) services, had consistent VADIR and VA Corporate Data Warehouse (CDW) data regarding their sex and age, and had a usable address listed in CDW. VA CDW and VADIR data were cross-referenced and used to construct the initial sampling frame, which included 14,194 eligible Veterans. From these, 3,000 women Veterans were randomly selected, stratified by region (Northeast, Midwest, South, and West) and age (tertiles: ≤35; 36–42; ≥43), and invited to participate.

Women in our sampling frame were sent three invitation mailings, unless they opted out of participating or completed the survey in response to an initial mailing. Mailings began in May 2020 and were spaced approximately eight weeks apart. The initial mailing included an invitation letter with the online survey link, a postcard consent form, paper survey, stamped addressed return envelope, debriefing form, and opt-out card. Subsequent mailings emphasized the online participation option and did not include the paper survey, return envelope, or opt-out card.

Because many of the survey topics (e.g., firearm access, military sexual trauma, suicidal thoughts and behaviors) are sensitive and thus may be underreported [26, 27], we provided participants with the choice to participate anonymously; this approach is associated with higher rates of reporting private and potentially sensitive information [28–30]. For participants who did not participate anonymously or decline compensation, $20 was provided. The Colorado Multiple Institutional Review Board and local VA Research and Development Committee approved this study. All participants provided informed consent to participate, although we obtained a waiver of documentation of consent (i.e., no signatures were obtained from participants).

Most of the 3,000 mailings sent (*n* = 2,606; 86.9%) were presumed delivered (i.e., were not returned as undeliverable). Of those, 576 (22.1%) consented to participate, the majority (*n* = 528; 92.0%) of whom were eligible to take part. Those who were ineligible were excluded due to currently being active status or full-time Guard or Reserve, currently being active duty, or not having deployed. For the current aims, we removed individuals from the analytic sample if they were: missing data for all the COVID-19 firearm questions (*n* = 22), declined to respond to all the COVID-19 firearm questions (*n* = 2), were missing the majority of their COVID-19 firearm data (*n* = 2), or had a combination of missing data and decline to respond responses to the COVID-19 firearm questions (*n* = 1). Thus, our analytic sample included 501 participants.

### Measures

#### Firearm beliefs and behaviors

To assess changes in women Veterans’ firearm-related *beliefs*, participants were asked if any of their beliefs about firearms had changed “since the beginning of the Coronavirus Disease 2019 (COVID-19) pandemic (since 3/11/2020),” with response options of yes, no, or decline to respond (S1 Appendix). Participants who responded affirmatively were asked to describe (free text) how their beliefs had changed.

To assess firearm-related *behaviors* during the pandemic, participants were asked if they had purchased a firearm, purchased ammunition, made household firearms more easily accessible, loaded firearms, began carrying a firearm, or engaged in other behaviors (with a free text prompt) since the beginning of the COVID-19 pandemic (S1 Appendix). Response options included yes, no, or decline to respond. Those who selected “yes” to any firearm-related behavior(s) were asked to describe their reason(s) for the change (free text response). Participants who responded affirmatively (“yes” response) to at least one firearm-related behavior during the pandemic (i.e., purchased a firearm, purchased ammunition, made household firearm(s) more easily accessible, loaded previously unloaded firearms, and/or began carrying a firearm; responses of “other” were excluded) were considered to have engaged in firearm behavior during the pandemic.

We also included questions to describe the sample with respect to current firearm access and ownership. For example, we assessed for personal firearm ownership (“Have you ever personally owned a gun?” with a response option that reflected current ownership) and if the participant lived with someone who owned a firearm (“Does anyone else you live with currently own any type of gun?”). Participants who responded affirmatively regarding current firearm ownership and/or currently living with someone who owned a firearm were considered to have household firearm access.

#### Demographics and military service

The survey also assessed participants’ demographic characteristics and military service history, which were analyzed for descriptive purposes (to describe the sample) and considered as potential covariates and/or predictor variables for quantitative analyses examining factors associated with changes in firearm beliefs and engagement in firearm behaviors during the pandemic.

#### Military Sexual Trauma

We administered the VA Military Sexual Trauma Screen, which includes two questions used to determine a history of military sexual trauma (i.e., affirmative response to at least one question): (1) “When you were in the military, did you ever receive unwanted, threatening, or repeated sexual attention (for example, touching, cornering, pressure for sexual favors, or inappropriate verbal remarks, etc.)?” [military sexual harassment] (2) “When you were in the military, did you have sexual contact against your will or when you were unable to say no (for example, after being forced or threatened or to avoid other consequences)?” [military sexual assault]

Response options for each question include the following: yes, no, or decline to respond. The VA MST Screen has been administered clinically to more than 4 million Veterans within the Veterans Health Administration [31], and construct validity has been established [32]. Nonetheless, given substantial heterogeneity in MST experiences and distinctions in outcomes and risk associated with military sexual harassment and sexual assault [33, 34], we analyzed item-level responses to ascertain MST type. Specifically, responses were scored to reflect the following mutually exclusive categories: none (did not report experiencing military sexual harassment or assault), military sexual harassment (reported experiencing military sexual harassment, but not military sexual assault), military sexual assault (reported experiencing military sexual assault, irrespective of responses regarding military sexual harassment). Participants’ most “severe” MST type was coded as “declined to respond” if they: declined to respond to both MST questions; declined to respond regarding military sexual harassment and denied experiencing military sexual assault; or declined to respond to the military sexual assault question.

#### Posttraumatic Stress Disorder (PTSD) symptoms

The PTSD Symptom Checklist for DSM-5 (PCL-5) [35] was administered to assess for recent PTSD symptom severity. The PCL-5 includes twenty items that directly align with DSM-5 PTSD symptoms. Items were rated on a 5-point Likert scale ranging from 0 (not at all) to 4 (extremely), with a potential total score range of 0 to 80; higher scores reflect greater PTSD symptom severity. Construct validity, internal reliability, and test-retest reliability have been well-established for the PCL-5, including with Veteran samples; scores of 31–33 are recommended as a screening threshold for a probable PTSD diagnosis [36].

#### Suicidal thoughts and behaviors

Lastly, to describe the sample, a modified self-report version of the Self-Injurious Thoughts and Behaviors Interview (SITBI [37]) was administered to assess for the most severe lifetime suicidal self-directed violence experienced (suicide attempt, suicidal ideation, or neither).

### Analytic plan

We used a concurrent nested mixed-method design [38], in which we collected qualitative and quantitative data simultaneously, and used our qualitative findings to guide and inform subsequent quantitative analysis.

#### Qualitative

To examine how women Veterans’ firearm beliefs changed during the COVID-19 pandemic, as well as their reasons for engaging in firearm behavior during the pandemic, we used an inductively driven thematic analysis of open-ended free text responses [39]. Three of the study co-authors examined all of the free text responses from participants who reported experiencing changes in their firearm-related beliefs or who reported engaging in firearm behavior during the pandemic. Prior to analyzing the qualitative data, team members engaged in bracketing [40, 41], reflecting on their personal expectations regarding the data, the basis of those assumptions, any potential biases, and how they would address these when analyzing and interpreting the data. Team members then independently reviewed and analyzed participants’ free text responses, documenting their individual impressions. Thereafter, they met to discuss potential themes generated from the data to reach consensus. Consensus was reached through ongoing discussions, iteratively deliberating and resolving differences in code applications and interpretations until there was agreement regarding themes. These routine and iterative coding meetings allowed for diverse perspectives on emergent themes and qualitative findings, further mitigating potential biases between qualitative coders and the larger research team.

Below we present de-identified quotes from participants that exemplify the themes identified. To provide context for the quotes reflecting women Veterans’ reasons for engaging in firearm behavior(s) during the pandemic, we present corresponding information from their self-reported responses regarding the specific firearm behaviors that they engaged in during the pandemic.

#### Quantitative

Quantitative analyses were conducted with SAS v9.4. To examine if data on firearm beliefs and behaviors during the COVID-19 pandemic were missing at random, we conducted t-tests and chi-square tests, as appropriate, to determine if missingness was associated with any of the variables of interest. In comparing characteristics of the overall sample to that of the subsample with missing data on COVID-19 firearm beliefs and behaviors, few significant differences were identified (data not shown), except that those missing data on the COVID-19 firearm-related questions were also likely to be missing data regarding lifetime suicidal self-directed violence; all other data appeared to be missing at random. However, those missing data on the COVID-19 firearm-related questions were also likely to be missing data at a high frequency (>10%) for other sensitive questions, including PTSD symptoms and general firearm access and ownership.

To examine the extent to which women Veterans reported changes in their firearm beliefs and reported engaging in firearm behaviors during the pandemic, *n*’s and percentages were calculated, with 95% Confidence Intervals (CIs). Descriptive statistics (*n*’s and percentages for categorical variables; means and standard deviations for continuous variables) were also calculated to describe the sample, overall and for the subsamples with and without self-reported changes in firearm beliefs and engagement in firearm behavior(s) respectively.

To identify factors associated with changes in firearm beliefs and engagement in firearm behavior(s) during the pandemic, log-binomial regression models examined changes in firearm beliefs and engagement in firearm behavior(s) during the pandemic as two separate outcomes of interest; prevalence ratios and 95% CIs are reported from these models. Selection of predictor variables to include in each model was guided by our qualitative findings and included the following: race (White, Black, or Other [due to small sample sizes for other groups]); marital status (married/cohabitating or other), minors present in the home (yes/no), urban residence (yes/no), history of military sexual trauma (none [neither military sexual harassment or assault reported], military sexual harassment, or military sexual assault), and PTSD symptom severity (continuous score). Covariates assessed as potential confounders included demographic variables (age, ethnicity, and education), military service characteristics (years since separation from military service, branch of service, deployments [single/multiple], combat zone deployments [yes/no]), and rank [enlisted or officer]), and most severe lifetime suicidal self-directed violence (none, suicidal ideation, or suicide attempt). Confounders to include in multivariate models were determined by conducting a series of chi-square and t-tests, as appropriate, to identify any variables significantly (*p* < .05) associated with both the predictor and outcome variables of interest for a given model. No variables met confounder criteria; thus, no covariates were included in the regression models (i.e., multivariate models were not fit). For all analyses, *p* < .05 was used to determine statistical significance. That is, we present all prevalence ratios with 95% CIs and *p*-values to allow readers to ascertain clinical and statistical significance [42, 43].

## Results

### Sample characteristics

Sample characteristics are in Table 1. Participants tended to be middle-aged (mean age = 42.62 years). Most participants identified as White (68.06%) and non-Hispanic (85.31%). A smaller portion of participants identified as Black (22.75%), Asian or Pacific Islander (4.39%), Native American or Alaskan Native (2.99%), or “Other” (3.99% who provided responses outside of the aforementioned categories). Less than half of the sample was currently married or cohabitating (57.00%), and nearly half (46.51%) had individuals under 18 years of age living in their home. Region of residence widely varied in terms of rurality and urbanicity. Most of the sample (81.84%) reported having an Associate degree or higher.

**Table 1:** Characteristics of the overall sample (*n* = 501).

Variable	Total Sample (*n* = 501)
	M (SD)
**Age (years)** ^a^^,^^b^	42.62 (10.31)
**Years since military separation** ^a^^,^^c^	7.48 (4.49)
**PTSD symptom severity (PCL-5)**	30.37 (22.16)
	**n (%)**
**Race** ^d^^,^^g^	
White	341 (68.06)
Black	114 (22.75)
Native American or Alaskan Native	15 (2.99)
Asian or Pacific Islander	22 (4.39)
Other	20 (3.99)
**Ethnicity** ^f^	
Hispanic	73 (14.69)
Non-Hispanic	424 (85.31)
**Marital status** ^g^	
Married/Cohabitating	285 (57.00)
Single	112 (22.40)
Widowed	4 (0.80)
Divorced/Separated	99 (19.80)
**Minors in home**	
None	268 (53.49)
Present	233 (46.51)
**Residence** ^e^^,^^h^	
Rural	78 (15.66)
Town (small or medium)	148 (29.72)
Suburb	134 (26.91)
City	138 (27.71)
**Education**	
High school or some college	91 (18.16)
Associate or Bachelors	263 (52.50)
Masters or doctorate	147 (29.34)
**Military branch** ^d^^,^^g^	
Army	262 (52.30)
Air Force	117 (23.35)
Navy/Coast Guard	111 (22.16)
Marine Corps	34 (6.79)
**Deployments**	
Single	242 (48.30)
Multiple	259 (51.70)
**Combat zone deployment(s)** ^i^	
Yes	433 (86.77)
No	66 (13.23)
**Rank**	
Enlisted	344 (68.66)
Officer	157 (31.34)
**Military sexual trauma (most severe)** ^a^	
None	167 (33.81)
Military sexual harassment	168 (34.01)
Military sexual assault	136 (27.53)
Declined to respond	23 (4.66)
**Suicidal self-directed violence—lifetime (most severe)** ^g^	
None	267 (53.40)
Suicidal ideation	159 (31.80)
Suicide attempt	74 (14.80)
**Current firearm access** ^j^^,^^k^	
Yes	288 (58.06)
No	208 (41.94)
**Current personal firearm ownership** ^e^	
Yes	222 (44.58)
No	276 (55.42)
**Current household ownership** ^k^	
Yes	178 (35.89)
No	318 (64.11)

M = mean; PCL-5 = PTSD Symptom Checklist for DSM-5; SD = standard deviation

^a^ Missing *n* = 7

^b^ Range = 24–76; Median = 40

^c^ Refers to years since separation from military service

^d^ Not mutually exclusive

^e^ Missing *n* = 3

^f^ Missing *n* = 4

^g^ Missing *n* = 1

^h^ Total excludes those who selected “Don’t Know” (*n* = 2)

^i^ Missing n = 2

^j^ Reported currently owning a firearm and/or living with someone who owned a firearm.

^k^ Missing *n* = 5

The most common branch of service was Army (52.3%). Over half of participants had multiple (51.70%) rather than single (48.30%) deployments, and the majority had been deployed to a combat zone (86.77%). The majority had been enlisted (68.66%).

Nearly two-thirds (61.54%) screened positive for military sexual trauma (reported a history of military sexual harassment and/or sexual assault). Specifically, of the full sample, relatively similar proportions reported no MST history (33.81%) or military sexual harassment as their most severe MST type (34.01%), and a slightly lower proportion reported experiencing military sexual assault (27.53%); a small portion declined to respond to the MST questions (4.66%). The mean PCL-5 score in the sample was 30.37 (SD = 22.16; median = 29.00), suggesting, on average, a level of PTSD symptoms within the sample close to the clinical threshold for screening positive for PTSD.

Regarding most severe lifetime suicidal self-directed violence experienced, 14.80% of participants reported a suicide attempt and 31.80% reported suicidal ideation as the most severe suicidal self-directed violence they had experienced. 58.06% of the sample reported current firearm access (i.e., owned a firearm and/or lived with someone who owned a firearm). Specifically, 44.58% of participants reported that they currently owned a firearm, and 35.89% reported currently living with someone else who owned a firearm.

### Prevalence of changes in firearm-related beliefs and behaviors

As reflected in Table 2, 13.88% (*n* = 69) and 22.15% of participants reported changes in their firearm-related beliefs and engagement in firearm behaviors, respectively, during the COVID-19 pandemic. Making household firearms more easily accessible was the most commonly reported behavior during the pandemic (16.13%), followed by purchasing ammunition (11.97%). Smaller percentages of participants reported that they began carrying a firearm more frequently (6.71%), loaded firearms that were previously stored unloaded (5.69%), or purchased a firearm (4.24%) (Table 2).

**Table 2:** Prevalence of changes in women Veterans’ firearm beliefs and engagement in firearm behaviors since the COVID-19 pandemic began.

	Yes			No		
	*n*	%	95% CI	*n*	%	95% CI
**Changed firearm beliefs** ^a^	69	13.88	10.96, 17.24	428	86.12	82.76, 89.04
**Engaged in firearm behaviors** ^b^	109	22.15	18.55, 26.09	383	77.85	73.91, 81.45
Made household firearm(s) more easily accessible ^c^	80	16.13	13.00, 19.67	416	83.87	80.33, 86.99
Purchased ammunition ^c^	59	11.97	9.23, 15.17	434	88.03	84.83, 90.77
Began carrying a firearm (or started carrying more often) ^c^	33	6.71	4.66, 9.30	459	93.29	90.70, 95.34
Loaded firearms that were previously unloaded ^c^	28	5.69	3.81, 8.13	464	94.31	91.87, 96.19
Purchased a firearm ^c^	21	4.24	2.64, 6.42	474	95.76	93.58, 97.36
Other	34	6.79	4.74, 9.36	467	93.21	90.64, 95.26

CI = confidence interval.

^a^ Excludes those who declined to respond (*n* = 4)

^b^ Indicated "yes" to any specific firearm-related behavior, aside from "other"; the percentage for the overall change in firearm behaviors variable excluded those with a combination of missing/declined responses and "no" responses (*n* = 9) for the individual firearm behavior questions.

^c^ Total n excludes those who declined to respond (*n* = 2) or who were missing data for the respective item.

Additionally, 6.79% of participants (*n* = 34; not included when calculating the 22.15% noted above) endorsed engaging in “other” behaviors during the pandemic. These included preparatory behavior to potentially obtain a firearm (“inquired”; “researched how to obtain a firearm”; “looking to purchase, but most stores have been closed. Otherwise I would have made purchases by now.”); acquiring or increasing the accessibility of other means of protection (“started carrying a knife”; “sleep with a baseball bat”; “I carry a blade with me while riding my bike”; “I bought a knife. It had been on my list to buy one since living on my own. But I only bought it after big stores were reopened.”); or increasing home security (“Just installed more cameras”; “I have implanted [sic] additional measures within my home to give me advanced warning of people (in addition to the cameras and alarms I already had)”.

In terms of overlap in responses to the firearm beliefs and behaviors questions, for those with non-missing data on both, 4.71% (*n* = 23) reported changes in both their beliefs and behaviors during the pandemic; 8.81% (*n* = 43) reported experiencing changes in their firearm beliefs, without engagement in firearm behaviors; and 17.42% (*n* = 85) reported engaging in firearm behaviors, but denied experiencing changes in their firearm beliefs.

Of the 109 participants who reported engaging in firearm behavior(s) during the pandemic, 97.25% (*n* = 106) reported that they currently had household firearm access, 88.99% (*n* = 97) reported currently personally owning a firearm, and 52.29% (*n* = 57) reported currently living with someone who owned a firearm.

### Thematic analysis

Desire for increased protection was an overarching theme in women Veterans’ reasons for changing their firearm-related beliefs and behaviors during the pandemic. Specifically, women Veterans described newly or increasingly believing that firearms were necessary and important for protection of oneself, one’s family, and one’s property against others. Specific circumstances contributing to this overarching theme of protection were detailed across three domains: (1) uncertainties brought on by the pandemic; (2) pandemic-related threats necessitating self-defense, preparedness, and self-sufficiency; and (3) political and social unrest, and racial justice demonstrations.

#### Pandemic personal protection: “*Everyone has one so it seems like I would need one too*”

Women Veterans who experienced changes in their firearm-related beliefs or who reported engaging in firearm behaviors during the pandemic described beliefs related to firearms becoming more necessary in the midst of the pandemic. They commonly described how firearms were “a necessary tool to have in the home for home safety purposes,” stating how “firearms are much needed today” and that “it’s more important to own a gun” now than before the pandemic. Detailing her thought process, one woman stated, “I was fully against owning one until I feared people may become aggressive. Owning a gun seems like a safe thing to own right now.” Uncertainties and fears during the early stages of the pandemic contributed to unease and heightened the perceived need for a firearm. “I was very worried to have a gun in my home,” one respondent professed, “but after all the recent events, I feel the need to have further protection.” Another woman felt similarly, expressing that despite her reservations regarding her own mental health concerns, she was considering acquiring a firearm, “I never wanted to own a firearm due to the responsibility they bring and my mental health. However, since COVID-19, I have been highly considering it.”

These shifts in beliefs surrounding firearm ownership and access amidst the pandemic, accompanied by a desire for personal protection, were further heightened by Veterans’ personal circumstances. Some women in our sample lived alone, with one describing how she “recently became single again. I have to protect myself at night.” Another Veteran shared, “living as a newly single mother with two young children, I want to feel safer.” This contributed to her applying for a concealed carry permit and planning to purchase a firearm. The perceived need to protect one’s children through firearm acquisition or firearm accessibility was common within our sample. One woman shared that she increased the accessibility of household firearms by loading previously unloaded firearm(s). She explained: “Society seems not to care about anyone’s safety and if anyone shows up to our home, I need to protect my kids.” The fear that individuals lack empathy in times of turmoil was expressed by women (especially single women), who noted their children as a reason for increasing their firearm accessibility. Having purchased a firearm and ammunition and having made household firearm(s) more accessible, one woman stated that, “People, as a group, react badly to things. I have to protect my [sic] and my baby because we live alone.”

#### Pandemic defense, preparedness, and self-sustainability: “*I feel like I should be more on guard and prepared to defend myself and family”*

In addition to the general need to protect oneself, borne largely out of fears of unknown circumstances that could arise from the pandemic, many women noted changes in their firearm beliefs and behaviors due to concerns over how others in society might react to these changes. Whether due to food shortages, supply chain disruptions, or a perceived need to acquire and preserve resources, women Veterans considered firearms to be necessary to protect themselves. “[I] feel like they are more needed due to some people going crazy over food availability, supplies of PPE’s [personal protective equipment] and gas or just anything. Some people don’t care and will put harm in your life for no reason at all.” Many detailed how firearms may be more necessary during the pandemic due to other people’s threatening reactions regarding scarcity of resources. One woman described how, “since the pandemic, others can be more on edge or do things they wouldn’t normally do, so I feel like I should be more on guard and prepared to defend myself and family.” Further highlighting concern regarding the potential for others to do harm to them, another woman remarked, “The virus has caused people to become irrational. I do not fear the virus nor believe in all the hype surrounding the virus. During times when necessities were scarce, I wanted to ensure my family and property were safe”.

Resource scarcity and the need to prepare for the worst should someone want to seize one’s limited resources was a common sentiment. Women detailed various concerns about home invasions, burglaries, or break-ins to seize supplies. “Things with COVID-19, especially in the beginning, got really ugly,” a woman Veteran detailed, “I felt there was a threat of someone breaking into my home to steal things like food, toilet paper, paper towels.” She later decided to go out and purchase more ammunition for her firearms. Another woman explained: “People are going crazy buying up everything, I had to make sure my personal property was protected. I had one firearm, I just purchased more for extra safety.” One woman described how her beliefs about firearms had changed since the pandemic began, including feeling threatened by people rioting and protesting: “People have lost jobs and lost income and are in need of basic necessities to survive…I think everyone should have a weapon for personal defense.” Other women declared that “people can act crazy when they believe the media or find food to be scarce,” expressed that “people may become dangerous, aggressive, and unpredictable in these times,” and indicated that “people could start feeling desperate and try to rob or steal in my area.” Women Veterans described these concerns as their reasons for changing their firearm practices, in order to “feel better prepared.” One Veteran in particular exclaimed how she “moved [firearms] closer to my bed, in case in the middle of the night, someone comes to my door uninvited!”

#### Pandemic unrest: “*I see a lot of rioting and protesting*, *and I feel threatened”*

In conjunction with the outbreak of the pandemic, women Veterans noted that civil unrest, protests, and rioting during 2020 resulted in changes in how they thought about and used their firearms. Many women in this sample felt that viewing and experiencing these instances of unrest raised their need to be cautious and made them reconsider their firearm beliefs and behaviors. For example, one woman indicated, “the civil unrest/riots concern me about basic public safety.” Similarly, another stated, “with all the rioting, I have kept my firearm with me more often.” Another woman Veteran changed her firearm practices “to protect my family from possible intruders. When the protests and riots are happening, there were threats of breaking into homes that were located in gated communities, which describes mine”.

Many linked these civil disturbances to direct changes in their firearm practices and behaviors. One woman mentioned that she changed the way she thought about using and owning firearms, “not because of COVID, but because of race relations, riots, and attacks on innocent people and businesses.” Echoing this sentiment, a woman who increased the accessibility of her firearms at home recalled that, “we have one household firearm that is now accessible since COVID started, but not because of COVID, per se. It’s more because of the general increase in crime and unrest since COVID and the racial protests began.” Another respondent listed the reasons for her multitude of firearm changes: “1. You can never have enough ammo 2. Accessibility: did it to be more prepared for civil unrest 3. Loaded all firearms to be more prepared for civil unrest. 4. Carry on my person every day because you never know what can happen.” She had bought ammunition, increased firearm accessibility around her home, loaded previously unloaded firearms, and started carrying a firearm more often in response to pandemic unrest.

A unique intersection of the need for personal protection amidst fears regarding civil unrest during the pandemic concerned race. For example, one woman Veteran had considered obtaining a firearm to protect herself and her family, “I thought maybe since everyone is so on edge that it might be a good idea to have one.” However, she described being fearful to own a firearm due to her race: “But because I am African American, I also think that I do not want one because nothing good could come of me using even if it is solely to protect myself and my spouse from intruders at home. So, I’m equally afraid to own a gun as I am not to own one. It is a stressful and also a life-changing decision.” Of import, she had “considered purchasing a firearm but have not gone beyond the thought process. I have not completed any research on firearms, that is to say.” Another woman Veteran who identified racially as Black expressed concerns about safety and rising racial tensions as the context for why she changed her firearm access: “During COVID-19, racist behavior has magnified and raised safety concerns for my household. My firearm is easier to access for protection of my home and family.”

### Factors associated with changes in firearm beliefs and engagement in firearm behaviors during the pandemic

Tables 3 and 4 present characteristics of the subsamples with and without reported changes in their firearm beliefs and engagement in firearm behaviors during the pandemic. Participants who reported changing their firearm beliefs (*t* = 3.08, *p* = 0.0028) and engaging in firearm behaviors (*t* = 2.20, *p* = 0.0293) during the pandemic reported significantly more severe current PTSD symptoms on the PCL-5. No other significant bivariate relationships were observed.

**Table 3:** Sample characteristics for participants with and without changes in their firearm beliefs during the COVID-19 pandemic.

Variable	Change in Firearm Beliefs (n = 69, 13.88%)	No Change in Firearm Beliefs (n = 428, 86.12%)	*p*-value^a^
	M(SD)		
**Age (years)** ^b^	43.55 (10.53)	42.45 (10.29)	0.7703
**Years since military separation** ^b^^,c^	7.13 (4.49)	7.53 (4.47)	0.5048
**PTSD symptom severity (PCL-5)**	38.00 (22.47)	29.06 (21.90)	0.0028
	**n(%)**		
**Race** ^d^^,^^e^			
White	52 (75.36)	287 (67.06)	0.1691
Black	12 (17.39)	101 (23.60)	0.2536
Native American or Alaskan Native	3 (4.35)	12 (2.80)	0.4492
Asian or Pacific Islander	1 (1.45)	21 (4.91)	0.3401
Other	1 (1.45)	19 (4.44)	0.3362
**Ethnicity** ^f^			0.9798
Hispanic	10 (14.71)	63 (14.82)	
Non-Hispanic	58 (85.29)	362 (85.18)	
**Marital status** ^g^			0.5252
Married/Cohabitating	37 (54.41)	247 (57.71)	
Single	19 (27.94)	91 (21.26)	
Widowed	1 (1.47)	3 (0.70)	
Divorced/Separated	11 (16.18)	87 (20.33)	
**Minors in home**			0.6408
None	39 (56.52)	229 (53.50)	
Present	30 (43.48)	199 (46.50)	
**Residence** ^e^^,^^h^			0.6709
Rural	14 (20.59)	64 (15.02)	
Town (small or medium)	129 (26.47)	129 (30.28)	
Suburb	17 (25.00)	116 (27.23)	
City	19 (27.94)	117 (27.46)	
**Education**			0.6508
High school or some college	10 (14.49)	80 (18.69)	
Associates or Bachelors	39 (56.52)	221 (51.64)	
Masters or doctorate	20 (28.99)	127 (29.67)	
**Military branch** ^d^^,^^g^			
Army	30 (43.48)	230 (53.74)	0.1133
Air Force	20 (28.99)	97 (22.66)	0.2507
Navy/Coast Guard	18 (26.09)	92 (21.50)	0.3939
Marine Corps	7 (10.14)	26 (6.07)	0.2076
**Deployments**			0.1085
Single	27 (39.13)	212 (49.53)	
Multiple	42 (60.87)	216 (50.47)	
**Combat zone deployment(s)** ^i^			0.4010
Yes	62 (89.96)	367 (86.15)	
No	7 (10.14)	59 (13.85)	
**Rank**			0.3714
Enlisted	44 (63.77)	296 (69.16)	
Officer	25 (36.23)	132 (30.84)	
**Military sexual trauma (most severe)** ^b^			0.0714
None	16 (23.19)	148 (35.15)	
Military sexual harassment	27 (39.13)	140 (33.25)	
Military sexual assault	25 (36.23)	111 (26.37)	
Declined to respond	1 (1.45)	22 (5.23)	
**Suicidal self-directed violence—lifetime (most severe)** ^g^			0.1419
None	32 (46.38)	232 (54.33)	
Suicidal ideation	29 (42.03)	129 (30.21)	
Suicide attempt	8 (11.59)	66 (15.46)	

M = mean; PCL-5 = PTSD Symptom Checklist for DSM-5; SD = standard deviation

^a^ Reflect chi-square or Fisher’s exact p-values for categorical variables and t-test *p*-values for age, years since military separation, and PCL-5.

^b^ Missing *n* = 7

^c^ Refers to years since separation from military service

^d^ Not mutually exclusive

^e^ Missing *n* = 3

^f^ Missing *n* = 4

^g^ Missing *n* = 1

^h^ Total excluded those who selected “Don’t Know” (*n* = 2)

^i^ Missing *n* = 2

For military sexual trauma, responses were coded as “declined to respond” for participants who: declined to respond to both MST questions; declined to respond regarding military sexual harassment and denied experiencing military sexual assault; or declined to respond to the military sexual assault question.

**Table 4:** Sample characteristics for participants with and without engagement in firearm behaviors during the COVID-19 pandemic.

Variable	Firearm Behaviors (n = 109, 22.15%)	No Firearm Behaviors (n = 383, 77.85%)	*p*-value ^a^
**Age (years)** ^b^	42.45 (9.77)	42.64 (10.52)	0.8609
**Years since military separation** ^b^^,^^c^	7.17 (4.33)	7.60 (4.56)	0.3629
**PTSD symptom severity (PCL-5)**	34.22 (21.02)	29.15 (22.13)	0.0293
**Race** ^d^^,^^e^			
White	74 (67.89)	260 (67.89)	0.9992
Black	25 (22.94)	88 (22.98)	0.9929
Native American or Alaskan Native	5 (4.59)	10 (2.61)	0.2897
Asian or Pacific Islander	2 (1.83)	19 (4.96)	0.1879
Other	6 (5.50)	14 (3.66)	0.3888
**Ethnicity** ^f^			0.3164
Hispanic	19 (17.76)	53 (13.87)	
Non-Hispanic	88 (82.24)	329 (86.13)	
**Marital status** ^e^			0.7199
Married/Cohabitating	67 (61.47)	214 (56.02)	
Single	20 (18.35)	88 (23.04)	
Widowed	1 (0.92)	3 (0.79)	
Divorced/Separated	21(19.27)	77 (20.16)	
**Minors in home**			0.6219
None	56 (51.38)	207 (54.05)	
Present	53 (48.62)	176 (45.95)	
**Residence** ^f^^,^^g^			0.3621
Rural	22 (20.18)	53 (13.95)	
Town (small or medium)	28 (25.69)	117 (30.79)	
Suburb	31 (28.44)	102 (26.84)	
City	28 (25.69)	108 (28.42)	
**Education**			0.6343
High school or some college	22 (20.18)	66 (17.23)	
Associates or Bachelors	53 (48.62)	205 (53.52)	
Masters or doctorate	34 (31.19)	112 (29.24)	
**Military branch** ^d^^,^^e^			
Army	65 (59.63)	193 (50.39)	0.0883
Air Force	24 (22.02)	92 (24.02)	0.6639
Navy/Coast Guard	18 (16.51)	89 (23.24)	0.1333
Marine Corps	9 (8.26)	24 (6.27)	0.4636
**Deployments**			0.4772
Single	56 (51.38)	182 (47.52)	
Multiple	53 (48.62)	201 (52.48)	
**Combat zone deployment(s)** ^h^			0.2414
Yes	98 (89.91)	326 (85.56)	
No	11 (10.09)	55 (14.44)	
**Rank**			0.7768
Enlisted	73 (66.97)	262 (68.41)	
Officer	36 (33.03)	121 (31.59)	
**Military sexual trauma (most severe)**			0.1457
None	27 (24.77)	137 (36.34)	
Military sexual harassment	42 (38.53)	126 (33.42)	
Military sexual assault	35 (32.11)	96 (25.46)	
Declined to respond	5 (4.59)	18 (4.77)	
**Suicidal self-directed violence—lifetime (most severe)** ^e^			0.9213
None	58 (53.21)	203 (53.14)	
Suicidal ideation	36 (33.03)	121 (31.68)	
Suicide attempt	15 (13.76)	58 (15.18)	

M = mean; PCL-5 = PTSD Symptom Checklist for DSM-5; SD = standard deviation

^a^ Reflect chi-square or Fisher’s exact p-values for categorical variables and t-test *p*-values for age, years since military separation, and PCL-5.

^b^ Missing *n* = 6

^c^ Refers to years since separation from military service

^d^ Not mutually exclusive

^e^ Missing *n* = 1

^f^ Missing *n* = 3

^g^ Total excluded those who selected “Don’t Know” (*n* = 2)

^h^ Missing *n* = 2

Next, log-binomial regression was used to further describe (in terms of prevalence ratios) associations between identified factors of interest and changes in firearm beliefs during the COVID-19 pandemic (Table 5). For every one-point increase in PTSD symptom severity, the prevalence of changes in firearm beliefs during the pandemic increased by 1.53%. Furthermore, we were able to detect additional associations based on the type of MST experienced. Specifically, a history of military sexual assault (relative to no MST) was associated with an 88.4% increase in the prevalence of changes in firearm beliefs during the pandemic. In contrast, significant associations were not detected for military sexual harassment, race, marital status, having minors in the home, or residing in an urban area.

**Table 5:** Log-binomial regression models examining factors associated with changes in women Veterans’ firearm beliefs during the COVID-19 pandemic.

Variable	n	PR	SE	95% CI	p
Race					
White*	324	--			
Black	113	0.6617	0.1992	0.3668, 1.1937	0.1701
Other	60	0.5192	0.2319	0.2164, 1.2460	0.1423
**Marital status **					
Married/cohabitating*	212	--			
Other	284	1.2224	0.2536	0.7208, 1.7477	0.0693
**Minors in home**	497	1.1108	0.2506	0.7139, 1.7284	0.6412
**Urban residence **	494	0.9797	0.2457	0.5993, 1.6016	0.9349
**Military sexual trauma** ^**a**^					
None*	164	--			
Military sexual harassment	167	1.6572	0.4901	0.9282, 2.9586	0.0876
Military sexual assault	136	1.8842	0.5623	1.0498, 3.3817	0.0338
**PTSD (PCL-5) **	497	1.0153	0.0049	1.0057, 1.0251	0.0018

No covariates met criteria for inclusion in these models. CI = confidence interval; PCL-5 = PTSD Symptom Checklist for DSM-5; PR = prevalence ratio; SE = standard error.

^a^ Those who declined to respond were excluded for this analysis.

*Reference category

Similar results were obtained in log-binomial regression analyses examining factors associated with engagement in firearm behaviors during the pandemic (Table 6). Again, significant associations were detected between both PTSD symptom severity and military sexual assault history (relative to no MST history) with engagement in firearm behaviors during the pandemic. For every one-point increase in PTSD symptom severity, the prevalence of engaging in firearm behaviors during the pandemic increased by 0.78%. Additionally, history of military sexual assault (relative to no MST) was associated with a 62.28% increase in the prevalence of engagement in firearm behaviors during the pandemic. Consistent with our findings regarding firearm beliefs, significant associations were not detected for race, marital status, having minors in the home, residing in an urban area, or military sexual harassment.

**Table 6:** Log-binomial regression models examining factors associated with women Veterans’ engagement in firearm behaviors during the COVID-19 pandemic.

Variable	n	PR	SE	95% CI	p
Race					
White*	319	--			
Black	113	0.9940	0.2040	0.6649, 1.4861	0.9767
Other	60	0.9735	0.2598	0.5770, 1.6423	0.9198
**Marital status **					
Married/cohabitating*	281	--			
Other	210	0.8388	0.1463	0.5960, 1.1806	0.3135
**Minors in home**	492	0.9200	0.1555	0.6606, 1.2812	0.6218
**Urban residence**	489	1.1145	0.2169	0.7611, 1.6321	0.5774
**Military sexual trauma** ^**a**^					
None*	164	--			
Military sexual harassment	168	1.5185	0.3354	0.9849, 2.3413	0.0586
Military sexual assault	131	1.6228	0.3696	1.0385, 2.5360	0.0335
**PTSD (PCL-5) **	492	1.0078	0.0037	1.0005, 1.0151	0.0352

No covariates met criteria for inclusion in these models. CI = confidence interval; PCL-5 = PTSD Symptom Checklist for DSM-5; PR = prevalence ratio; SE = standard error.

^a^ Those who declined to respond were excluded for this analysis.

*Reference category

## Discussion

From 2001 to 2019, use of firearms as a method of suicide increased more among women Veterans (by 12.8%) than for non-Veteran women (4.2.% decrease) and Veteran men (2.9% increase) [2]. Moreover, women have been disproportionately affected by the pandemic–for example, with respect to caregiving responsibilities, gender-based violence, employment, more limited access to healthcare services (e.g., reproductive healthcare), and daily life [44–46]. Thus, there has been a particular need to understand how the prevalence of suicide risk factors may have changed among women during this time period [47]. As firearm access is a risk factor for suicide [7, 8], we conducted a mixed-methods analysis to understand changes in firearm beliefs and engagement in firearm behaviors during the early pandemic era among previously deployed post-9/11 era women Veterans.

A substantial portion of women Veterans in our sample described experiencing changes in their firearm beliefs (13.88%) and engaging in (any) firearm behaviors (22.15%) from mid to late 2020. In particular, women Veterans described increasingly believing that firearms were necessary for protecting themselves and their families. Of note, this was identified among some women who previously felt that firearms were unsafe to own. Potentially as a result of increased beliefs that firearms are necessary for protection, over one-fifth of women Veterans in our sample engaged in behaviors that potentially increased their access to firearms, such as acquiring new firearms or changing how existing firearms were stored. This is generally consistent with another study investigating firearm use during the COVID-19 pandemic, in which protection from others was the most common reason reported for purchasing a firearm in response to the pandemic [15]. Notably, approximately two-thirds of American firearm owners, including Veterans, reported that protection is their primary reason for firearm ownership, even prior to 2020 [48, 49].

Our finding that the perceived need for protection was prominent in women Veterans’ descriptions of the changes in their firearm beliefs and reasons for engaging in firearm behaviors during the pandemic also mirrors research from women Veterans prior to the pandemic [50]. However, there were differences from prior research with women Veterans in the specific circumstances that were described as prompting their perceived need for protection. Within the current sample, firearm behavior during the pandemic appeared to be driven by concerns about unpredictable, aggressive, or uncharacteristic behavior from others stemming from, for example, supply shortages or civil unrest. Our qualitative analyses suggested that being single, living alone, and having children also appeared to be salient contextual factors relevant to women Veterans’ safety-related concerns and firearm behaviors during the pandemic. However, neither marital status/cohabitation nor having minors at home was significantly associated with changes in firearm beliefs or engagement in firearm behaviors in our quantitative analyses.

Race was also identified as a relevant contextual factor within our qualitative analyses, though with mixed impact. Some women Veterans who identified as Black described perceiving an increased need to protect themselves and their families due to their race, yet were concerned that acquiring a firearm would introduce new risks into their household. Other women in our sample pointed to racial justice demonstrations as their reason for changing their firearm behaviors to protect themselves. Thus, for some women Veterans, firearms appeared to be perceived as potentially protective or risky depending on their race. These nuances may explain why race was not significantly associated with changes in firearm beliefs or engagement in firearm behaviors in our quantitative analyses–it may be that moderators are important to consider in future research given the complexity of these findings.

While our qualitative findings suggest that individual life circumstances (e.g., living alone, being single, having children in the home) potentially contributed to firearm beliefs and behaviors during the pandemic, our quantitative findings suggest that military sexual assault history and PTSD symptom severity are important to consider; both military sexual assault history and PTSD symptoms were associated with a higher prevalence of changes in firearm beliefs and engagement in firearm behaviors during the COVID-19 pandemic. We are unaware of other studies that have examined the roles of sexual violence or PTSD in firearm beliefs or behaviors during the pandemic. However, in research conducted with women Veterans prior to the pandemic, sexual assault during military service appeared to precipitate women Veterans’ firearm acquisition and firearm storage (e.g., keeping firearms easily accessible) following their military service [50]. Military sexual harassment has also been associated with increased prevalence of household firearm access among women Veterans [51]. The present findings add to this knowledge base by suggesting that post-9/11 women Veterans who experience military sexual trauma (specifically, sexual assault) have a higher prevalence of changed beliefs regarding firearms and a higher prevalence of engagement in firearm behaviors during the COVID-19 pandemic, relative to women Veterans who have not experienced military sexual harassment or assault. As survivors of military sexual trauma, particularly sexual assault, are already at elevated risk for suicidal thoughts and behaviors [52], these findings underscore the import of ensuring that lethal means safety initiatives include sufficient focus on firearms among women Veteran military sexual trauma survivors and that trauma-informed approaches are considered when doing so [53]. Further information relevant to this topic is available elsewhere [53–56].

Taken together, our findings suggest that a portion of post-9/11 women Veterans have increased their access to firearms during the COVID-19 pandemic, due to their perceptions of pandemic-related circumstances (e.g., others’ behavior in response to supply shortages and unemployment) or other concurrent events (e.g., protests, rioting). From a public health perspective, these findings warrant consideration given that the presence of household firearms is associated with increased risk for firearm mortality to household members, including children [57]. Our findings should also be considered in relation to co-occurring increases in risk factors for suicide since the pandemic began [34, 58], including interpersonal violence, financial stressors, caregiver burden, job loss, and decreased access to healthcare [44]. Further, there is initial evidence to suggest that women have been more likely to experience depression, anxiety, and posttraumatic stress disorder during this time period [44, 59–61]. In the presence of these risk factors, access to lethal means, such as firearms, could distinguish who engages in suicidal self-directed violence.

Notably, what may have initially been intended as a temporary, reactionary change in firearm access during the pandemic may become a long-term change for some. If that happens, elevated suicide risk due to increased access to lethal means may persist even for years after pandemic-era stressors resolve [62]. As such, timely efforts to address these changes in firearm behaviors in both public health and clinical suicide prevention initiatives is critical to addressing both current and future suicide risk, particularly among subsets of the women Veteran population who are already at increased chronic risk for suicide due to specific risk factors (e.g., military sexual assault history). Understanding prior considerations or perceptions about firearm ownership may be important for identifying how best to intervene in the future. For example, some respondents in our sample appeared to previously believe firearms were too risky to own.

While multi-faceted suicide prevention efforts are essential, our findings suggest that assessing for access to lethal means of suicide–including firearms–as part of suicide risk assessment is particularly important in the context and aftermath of the COVID-19 pandemic. Furthermore, considering that a substantial portion of women Veterans described engaging in behaviors that increased the accessibility of their firearms, emphasis on initiatives to safely store household firearms [63–65] is likely even more important during and following the COVID-19 pandemic. Given the salience of fear and the perceived need for protection in driving women Veterans’ firearm behaviors during the pandemic, identifying ways to assuage such fears and facilitate safety and protection in the context of recent national and global events is essential. Standardized lethal means safety counseling is now a mandated clinical practice within the VA [66]. As such guidance was not mandated at the time of data collection, it is possible that women Veterans’ firearm access and beliefs have changed since then, particularly for those who have used VHA services (data indicate, however, that only 14% of those using VHA services recall ever having a conversation with a provider about firearms [67]). Additionally, considering the prevalence of military sexual trauma in this sample, as well as in other studies of women Veterans with firearm access [51], a trauma-informed approach to firearm discussions with women Veterans may be particularly important [53, 56]. This may entail carefully attending to power differentials in the context of such discussions, clearly communicating the rationale for firearm questions and recommendations, and approaching firearm-related conversations collaboratively [68]. Additionally, in having conversations about firearms, it may be particularly important to empathically acknowledge that considering changing one’s firearm access (even if temporarily) may cause individuals to experience distressing emotions (e.g., anxiety) or thoughts (e.g., regarding safety), particularly if firearm access has served a protective function due to prior stressful or traumatic events [24, 53, 56]. Consequently, the patient and provider can work together to find ways to address any such emotions and beliefs while also working collaboratively to address firearm access in the context of suicide risk.

While specific methods of integrating these findings into clinical practice require additional research, prior work can provide some guidance. Research among women Veterans, including those with access to firearms, suggests that trust and rapport may be critical to facilitating discussions of firearm access and suicide risk, as well as shifting firearm storage practices [69]. In addition, providers can consider assessing the Veteran’s stage of change and appropriately matching motivational interviewing approaches to help Veterans in storing their firearms safely [70]. For example, in the presence of ambivalence to changing firearm access, providers can consider validating the emotional distress associated with fears regarding safety and protection during the COVID-19 pandemic, while also exploring the veracity of underlying beliefs. In addition, exploration of short- and long-term benefits and drawbacks to maintaining these behaviors (e.g., increased sense of safety, but potential for injury to a child or household adult) may be warranted.

Limitations of this study include the inability to differentiate between those who acquired household firearms for the first time versus firearm owners who acquired additional firearms. Given the eligibility criteria, our findings may not be generalizable to other populations. Additionally, our sample was specific to women Veterans; as such, gender differences could not be examined. It is similarly important to note that although the survey included empirically-validated measures, items used to assess changes in firearm beliefs and behaviors during the COVID-19 pandemic were developed specifically for this study and thus lacked prior validation. Also, rather than longitudinally assessing changes over time, we assessed self-reported change in beliefs and behaviors, which may be subject to recall bias. In addition, our overall response rate was low (22.1%), although similar to other national surveys of women Veterans (e.g., rates ranging from 17.9% to 19.6% [71, 72]); due to the option to participate anonymously, it is not possible to determine if differences were present between those who did and did not participate. We also could not examine if responses differed between early and late survey responders. Finally, it is worth noting that data were collected in the beginning of the COVID-19 pandemic (2020), and it is unclear how women Veterans’ firearm beliefs and behaviors might have changed following 2020, nor whether the pandemic itself or other relevant societal events (e.g., racial justice protests, political violence) were the predominant drivers of perceptions among any individual participant.

Importantly, although we did not examine the prevalence of first-time firearm ownership within this sample, the coalescence of data suggesting an increase in first-time firearm purchases (potentially spurred by changes in beliefs regarding firearm ownership) during this pandemic period [73], combined with shelter-in-place and lockdown orders that may have prevented many of these owners from attending firearm safety courses at local retailers or shooting ranges [16], may result in new owners lacking requisite knowledge and insights into means of preventing firearm injury or death, including suicide. However, additional data suggest that, while new firearm owners may lack training or expertise, they were more likely to safely store firearms than prior firearm owners who purchased new firearms [15]. Future research is warranted to determine if women Veterans who obtained firearms in the context of the pandemic differ regarding their willingness to discuss firearms with clinicians or in their preferences for firearm injury prevention initiatives. Such information would be expected to contribute to understanding regarding how to tailor suicide prevention efforts for post-9/11 women Veterans who have engaged in various firearm-related behaviors during the pandemic. Additionally, research aimed at better understanding the long-term impact of engaging in firearm behaviors during the COVID-19 pandemic may be informative.

## Supporting information

S1 Appendix COVID-19 items. COVID-19 firearm beliefs survey items.
(DOCX)
