# Supplementary material for: “Feel the need to prepare for Armageddon even though I do not believe it will happen”: Women Veterans’ Firearm Beliefs and Behaviors during the COVID-19 Pandemic, Associations with Military Sexual Assault and Posttraumatic Stress Disorder Symptoms
Source: PLoS One. 2023 Feb 10;18(2):e0280431. doi: 10.1371/journal.pone.0280431 (PMC9917279; doi:10.1371/journal.pone.0280431)
Supplement: S1 Appendix — COVID-19 firearm beliefs survey items. (DOCX) [file pone.0280431.s001.docx]

**1. Have any of your beliefs about firearms changed since the beginning of the Coronavirus Disease 2019 (COVID-19) pandemic (since 3/11/2020)?**

**No Yes Decline to respond**

***If “yes,” please describe:***

**2. Have you done any of the following since the beginning of the COVID-19 pandemic (since 3/11/2020)?**

|  | **Yes** | **No** | **Decline to respond** |
| --- | --- | --- | --- |
| a. Purchased a firearm |  |  |  |
| b. Purchased ammunition |  |  |  |
| c. Made household firearm(s) more easily accessible (e.g., storing them in ways that are easier to access quickly) |  |  |  |
| d. Loaded firearms that were previously unloaded |  |  |  |
| e. Began carrying a firearm (or started carrying a firearm more often) |  |  |  |
| g. Other, please describe: |  |  |  |

***3. If you selected “yes” to any of the items in Q2a-g, please describe your reason(s) for doing so.***
